# The importance of proper crystal-chemical and geometrical reasoning demonstrated using layered single and double hydroxides

**DOI:** 10.1107/S205251921300376X

**Published:** 2013-02-26

**Authors:** Ian G. Richardson

**Affiliations:** a School of Civil Engineering, University of Leeds, Leeds LS2 9JT, England

**Keywords:** atomistic modelling techniques, X-ray powder diffraction, layered double hydroxides

## Abstract

Atomistic modelling techniques and Rietveld refinement of X-ray powder diffraction data are widely used but often result in crystal structures that are not realistic, presumably because the authors neglect to check the crystal-chemical plausibility of their structure. The purpose of this paper is to reinforce the importance and utility of proper crystal-chemical and geometrical reasoning in structural studies. It is achieved by using such reasoning to generate new yet fundamental information about layered double hydroxides (LDH), a large, much-studied family of compounds. LDH phases are derived from layered single hydroxides by the substitution of a fraction (*x*) of the divalent cations by trivalent. Equations are derived that enable calculation of *x* from the *a* parameter of the unit cell and *vice versa*, which can be expected to be of widespread utility as a sanity test for extant and future structure determinations and computer simulation studies. The phase at *x* = 0 is shown to be an α form of divalent metal hydroxide rather than the β polymorph. Crystal-chemically sensible model structures are provided for β-Zn(OH)_2_ and Ni- and Mg-based carbonate LDH phases that have any trivalent cation and any value of *x*, including *x* = 0 [*i.e.* for α-*M*(OH)_2_·*m*H_2_O phases].

## Abstract 2

The importance and utility of proper crystal-chemical and geometrical reasoning in structural studies is demonstrated through the consideration of layered single and double hydroxides. New yet fundamental information is provided and it is evident that the crystal chemistry of the double hydroxide phases is much more straightforward than is apparent from the literature.

## 1. Introduction   

Gibbs *et al.* (2009 ▶) stated recently that ‘… *if mineralogists and geochemists persist in their study of minerals, their properties and relationships within the framework of empirical parameters like ionic radii, bond strength and electrostatic potential and forces and do not include first-principles quantum mechanical calculations and the study of ED distributions, then it* (is) *questionable whether our understanding of the crystal chemistry and the properties of minerals in their natural environments will advance much beyond that of* (the) *last century*’ (ED = electron density). Their view is no doubt correct, but unfortunately the proper use of crystal-chemical and geometrical reasoning – the approach of the ‘*last century*’ – is being neglected increasingly, with the result that much effort is wasted on unrealistic crystal structures. Such neglect is well illustrated by an atomistic modelling study published recently by Pellenq *et al.* (2009 ▶) in the *Proceedings of the National Academy of Sciences*. Pellenq *et al.* claim to have developed a ‘*realistic molecular model*’ for calcium silicate hydrate that is the main binding phase in most concrete (known as ‘C—S—H’), which is an important subject given that concrete is used on a vast scale (approximately 7 billion m^3^ per year worldwide; Gartner, 2004 ▶). Their model – which is derived from the structure of 14 Å tobermorite – has been used in a number of derivative works (*e.g.* Ji *et al.*, 2012 ▶) and the paper is already well cited. Yet inspection of their equilibrium structure reveals that it is unrealistic in light of what is known about the crystal chemistry of calcium silicates. In particular, a large proportion of the Ca—O distances in their model are either shorter than the minimum that is calculated from known structures of calcium silicate hydrates and related phases, or longer than the maximum distance, and more than half the Ca atoms in the model (which has 99 unique Ca atoms) are coordinated to fewer than six O atoms (their model has Ca in five-, four- and even threefold coordination: inspection of the structures of 34 crystalline calcium silicate hydrates and related phases – with a total of 132 unique Ca atoms – shows that six- and sevenfold are the ‘natural’ coordination for calcium cations in these phases, with none coordinated to fewer than six O atoms). The problem that Pellenq *et al.* attempted to address is a difficult one because C—S—H is compositionally and structurally very variable (Richardson, 2008 ▶); nevertheless, that is no excuse for the publication of a structure that is so unrealistic. The now-routine use of Rietveld refinement of X-ray powder diffraction (XRD) data can have similar consequences if scant consideration is given to the crystal-chemical plausibility of the resulting structure. The purpose of this paper is to reinforce the importance and utility of proper crystal-chemical and geometrical reasoning. It is achieved by using such reasoning to generate new yet fundamental information about layered double hydroxides (LDH), a large, much-studied family of compounds, members of which are involved in a diverse range of applications – including in catalysis and medicine and as anion exchangers (Cavani *et al.*, 1991 ▶) – and that are also important in the built environment (Taylor, 1997 ▶). Much has been written about LDH phases – including some highly cited review articles (*e.g.* Cavani *et al.*, 1991 ▶; Braterman *et al.*, 2004 ▶; Evans & Slade, 2006 ▶; Forano *et al.*, 2006 ▶; Mills, Christy, Genin *et al.*, 2012 ▶) – and so it is perhaps surprising to find that such a large and important family of compounds could be studied so extensively for over half a century without crystal-chemical and geometrical arguments being pursued more effectively. This paper provides such a treatment, which also necessitates a consideration of aspects of the crystal chemistry of layered divalent metal hydroxides because LDH phases are derived from them by the substitution of a fraction (*x*) of the divalent cations by trivalent cations. It is shown that it is possible to calculate *x* from the *a* parameter of the unit cell and *vice versa*, whichever is known with most confidence. The fact that *x* can be calculated from the *a* parameter provides a sanity test for the results of extant and future computer-simulation studies and crystal structure determinations using Rietveld analysis of powder XRD patterns. Model structures are provided that are crystal-chemically sensible for Ni- and Mg-based LDH phases that have any value of *x* (that is consistent with experiment), seemingly any trivalent cation, and that have carbonate as the charge-balancing interlayer ion. The models address for LDH phases the point raised by Woodley & Catlow (2008 ▶) that the challenge posed by crystal-structure prediction is ‘*… one of obtaining approximate models for unit-cell structures which may then be subsequently refined by methods using lattice energy or electronic structure techniques*’.

The procedure developed in this paper to calculate *x* from *a* simply involves consideration of the geometry of the metal–oxygen octahedra in the main layer of LDH phases and the determination of values for ionic radii that are sufficiently precise for bond-length calculations. The required information for both is obtained by extensive collation and analysis of literature data.

## 2. Geometrical relationships in the octahedral layer of single and double hydroxides   

**Figure 1:**
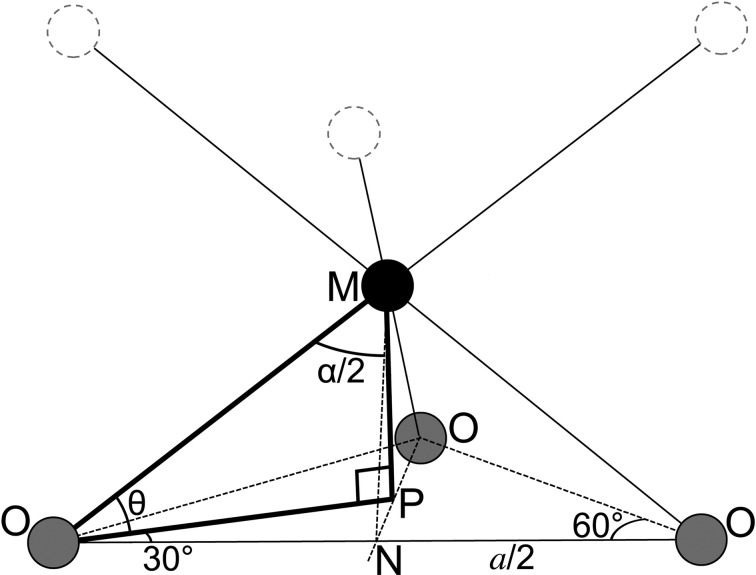
Geometry of an octahedron in the main layer of layered single and double hydroxides. The original projection was viewed along **b** with **c** up the page but for illustrative purposes it has been rotated around the **c** axis by ∼ 25° and tilted down by ∼ 10°. The circles labelled ‘O’ represent the O atoms of hydroxyl ions at the vertices of the octahedron and the circle labelled ‘*M*’ is the metal ion at the centre.

The β polymorphs of divalent metal hydroxides have the CdI_2_-type structure, which is trigonal, space group 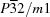 (No. 164). It is a eutactic structure (O’Keeffe, 1977 ▶) that consists of a hexagonal array of pseudo-close packed anions with octahedral sites between the anions that are alternately empty or occupied by metal cations and as a consequence it is layered. The main layer in the β-*M*(OH)_2_ phases consists of edge-sharing octahedra that have O atoms of hydroxyl ions at the vertices and a divalent metal cation at the centre. The edges of each octahedron are shared with six neighbours and each hydroxyl ion is shared by three octahedra, so the layers are electroneutral. Some geometrical relationships in the octahedron are illustrated in Fig. 1 ▶. A black circle labelled ‘*M*’ represents the site occupied by the metal cation, which is at the centre of the octahedron. The circles at its vertices represent O-atom sites that form the top and bottom of the octahedral layer. Both sets are shown but for clarity only the lower three circles are shaded and labelled ‘O’. These three sites form the triangular base of a squashed tetrahedron with the cation site, *M*. The base of the tetrahedron is an equilateral triangle and the length of a side of this triangle is equal to the *a* parameter of the unit cell. The distance between metal cations, *d*(*M*—*M*), is the same as the distance between oxygen sites that are in the same basal plane, *d*(O—O)^SP^ (*i.e.* that form either the top or bottom of the octahedral layer) and it is therefore also equal to the *a* parameter of the unit cell. It can be seen from inspection of Fig. 1 ▶ that *d*(O—O)^SP^ and so *d*(*M*—*M*) is related to the distance between the metal ion and the O atoms of the hydroxyl ions, *d*(*M*—O), by equation (1) (Brindley & Kao, 1984 ▶)
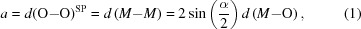
where α is the angle between two oxygen sites and the metal site when the two O atoms are in the same basal plane (*i.e.* ∠O*M*O on Fig. 1 ▶; ∠ 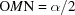). The distance between oxygen sites that are in opposite basal planes, *d*(O—O)^OP^, is only the same as between those in the same basal plane if α = 90°, *i.e.* for a regular octahedron, as shown by equation (2)

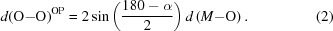
If the ions are considered to be packed as hard spheres, the cation in the CdI_2_-type structure must be ‘in contact’ (West, 1984 ▶) with the anions and so for β-*M*(OH)_2_ phases the distance between the divalent metal cation and the O atoms of the hydroxyl ions, *d*(*M*—O), is equal to the sum of the effective ionic radii
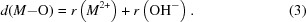
So
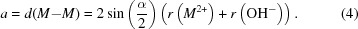
Since it is a eutactic structure the O atoms of the hydroxyl ions are not in contact with one another and so 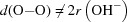; the same is more obviously also true for the metal cations, *i.e.* 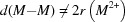.

**Figure 2:**
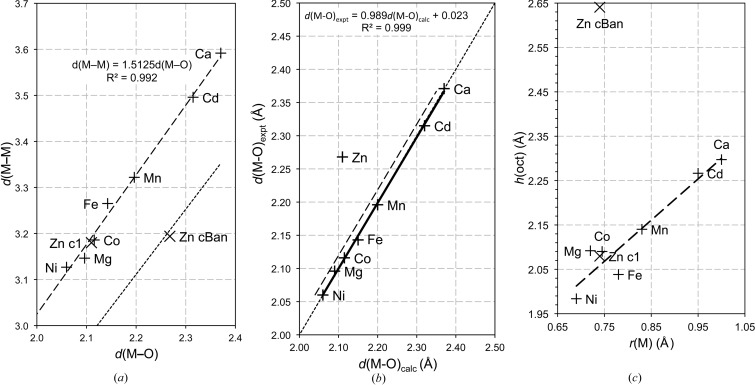
Distances in divalent metal hydroxides that have the CdI_2_-type structure. (*a*) Experimentally determined values of *d*(*M*—*M*) against *d*(*M*—O). (*b*) *d*(*M*—O) determined by experiment against the value calculated using ionic radii. (*c*) *h*(oct) against *r*(*M*). The data are for Mg(OH)_2_ (Catti *et al.*, 1995 ▶; Černý *et al.*, 1995 ▶; Chakoumakos *et al.*, 1997 ▶; Desgranges *et al.*, 1996 ▶; Isetti, 1965 ▶; Zigan & Rothbauer, 1967 ▶), Ni(OH)_2_ (Kazimirov *et al.*, 2010 ▶), Co(OH)_2_ (Pertlik, 1999 ▶), Fe(OH)_2_ (Parise *et al.*, 2000 ▶), Mn(OH)_2_ (Christensen & Ollivier, 1972 ▶), Cd(OH)_2_ (Bertrand & Dusausoy, 1970 ▶), Ca(OH)_2_ (Busing & Levy, 1957 ▶) and Zn(OH)_2_ (Baneyeva & Popova, 1969 ▶). There are two data points for β-Zn(OH)_2_: one calculated from Baneyeva & Popova’s structure that is labelled ‘Zn cBan’ and a second from this work that is labelled ‘Zn c1’.

The distance between the two planes of O atoms that form the octahedral layer, *h*(oct) – *i.e.* the thickness of the main layer – is twice the distance from the metal ion to the plane of O atoms, *i.e.* 2 × *d*(*M*—P) in Fig. 1 ▶. It is evident from inspection of Fig. 1 ▶ that *h*(oct) and *d*(*M*—O) are related by equation (5) (Brindley & Kao, 1984 ▶)
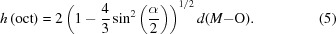
Substituting for *d*(*M*—O) from equation (3) gives
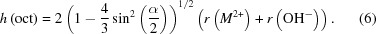
For a regular octahedron, the bond angle α is 90° and so the *a* parameter is given by equation (7), 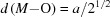, and 
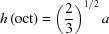


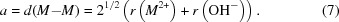
Fig. 2 ▶(*a*) is a plot of experimentally determined values of *d*(*M*—*M*) against *d*(*M*—O) for the eight β-*M*(OH)_2_ phases. The dotted line is for a regular octahedron (*i.e.* α = 90°; 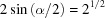) and so it is clear that the octahedron is close to ideal in only one of the structures, β-Zn(OH)_2_. There is a good linear relationship for the other seven phases, which indicates that they have a value for α that is approximately the same. From equation (1) the slope of the regression line is equal to 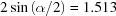 and so α = 98.3° (± 0.7) and the octahedron is as a consequence squashed in those seven structures. Brindley & Kao (1984 ▶) presented a similar figure but used values of *d*(*M*—O) that were calculated using ionic radii rather than those determined by experiment. The data point for β-Zn(OH)_2_ is clearly anomalous (the structure is from Baneyeva & Popova, 1969 ▶) and whilst an approximate value for its *a* parameter can be calculated using equation (4) (using α = 98.3° and the effective ionic radii of the Zn^2+^ and OH^−^ ions), the value of *a* is actually very sensitive to the value of α for the level of precision that is necessary and an accurate value for the effective radius of the O atom of the hydroxyl ion is also needed.

## 3. Determination of the effective radius of the O atom of the hydroxyl ion in divalent metal hydroxides   

Ionic radii vary with valence and coordination number and the relevant values for β-*M*(OH)_2_ phases are those for the divalent metal ion in sixfold coordination and the O atom of the hydroxyl ion in fourfold. The values used in this work are Shannon’s (1976 ▶) effective ionic radii (*i.e.* from the column labelled ‘IR’ in Table 1 of Shannon, 1976 ▶). The hydroxyl ion is non-spherosymmetrical (Oswald & Asper, 1977 ▶) and so Shannon’s value of 1.35 Å for *r*(OH^−^(IV)) corresponds to the radius of the O atom. Since the exact shape of the hydroxyl ion will vary (it is often referred to as ‘egg-shaped’ *e.g.* Zigan & Rothbauer, 1967 ▶; Brindley & Kao, 1984 ▶) Shannon’s value will not be appropriate for all bond-length calculations. The value of *r*(OH^−^(IV)) for the β-*M*(OH)_2_ phases can be determined by comparing the values of *d*(*M*—O) determined by experiment with those calculated using equation (3). The data for the eight β-*M*(OH)_2_ phases are plotted in Fig. 2 ▶(*b*), which shows that there is almost perfect agreement between the calculated and experimentally determined values of *d*(*M*—O) when a value of 1.370 Å is used for *r*(OH^–^(IV)) (when the Zn hydroxide is excluded): the dotted line in the figure represents perfect agreement, the crosses show the positions of the data points when *r*(OH^−^(IV)) = 1.370 Å is used in the calculation and the full line is the linear regression fit with *r*(OH^−^(IV)) = 1.370 Å. The dashed line shows the position of the fit when Shannon’s (1976 ▶) value for *r*(OH^−^(IV)) is used (*i.e.* 1.35 Å), which is clearly not appropriate.

## 4. Crystal structure of β-Zn(OH)_2_   

**Figure 3:**
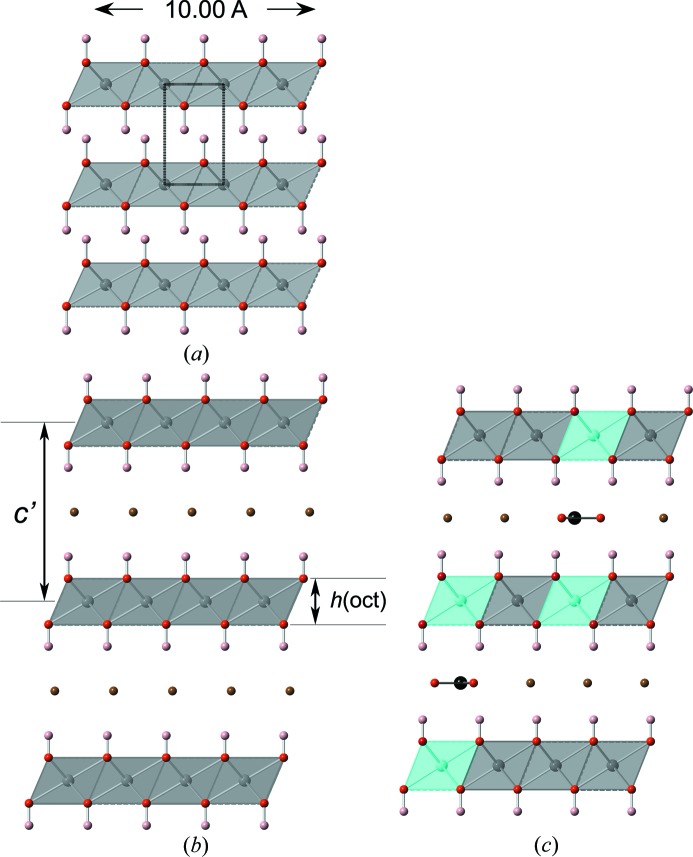
Polyhedral structure representations. (*a*) The model structure for β-Zn(OH)_2_ developed during this work. (*b*) *M*(OH)_2_·H_2_O, *i.e.* α-*M*(OH)_2_. (*c*) An LDH phase with *x* = 0.333. The spheres represent *M* ^2+^ (grey), *M* ^3+^ (blue), O^2−^ (red), C^4+^ (black), H^+^ (pink) and the O atoms of water molecules (brown). Lattice parameters and atomic coordinates are given in Table 1 ▶ for the β-Zn(OH)_2_ and Tables 2 ▶ and 3 ▶ for the model LDH phases. All structures are drawn to the same scale (a scale bar is inset in Fig. 3 ▶ *a*). The structures are all viewed along **b** with **c** up the page. A unit cell for β-Zn(OH)_2_ is outlined in Fig. 3 ▶(*a*): the length of the vertical line is *c* and the horizontal line is *a* cos (30). For the LDH phases, the layer spacing, *c*′, and the thickness of the main layer, *h*(oct), *i.e.* the distance between the two planes of O atoms that form the octahedral layer, are indicated in Fig. 3 ▶(*b*).

The data point in Figs. 2 ▶(*a*) and (*b*) for Baneyeva and Popova’s structure for β-Zn(OH)_2_ is clearly anomalous, suggesting strongly that there is a problem with the structure, as noted previously by Pertlik (1999 ▶) [Pertlik noticed that the *z* parameter for the O1 atom in the asymmetric unit for β-Zn(OH)_2_ is much larger than for other divalent metal hydroxides]. The experimental data reported by Baneyeva and Popova are not very precise (the *d* spacings are given to just two decimal places), but it is nevertheless straightforward to improve on their crystal structure simply by making it consistent with the other β-*M*(OH)_2_ phases. Inspection of their data (that are reproduced in Table 1 ▶, column *d*_exp_) shows that *d*_001_ = 4.69 Å and *d*_110_ = 1.59 Å, and so *c* = 4.69 Å and *a* = 2 × *d*_110_ = 3.18 Å. Substitution of *a* = 3.18 Å, *r*(Zn^2+^(VI)) = 0.74 Å (from Shannon, 1976 ▶) and *r*(OH^−^(IV)) = 1.370 Å (from Fig. 2 ▶ *b*) into equation (4) gives α = 97.80°, which is similar to the values for the other phases. Substitution of α = 97.80° and *r*(OH^−^(IV)) = 1.370 Å into equation (6) gives a value for the thickness of the main layer that is similar to the values for the other β-*M*(OH)_2_ phases, as shown in Fig. 2 ▶(*c*). The *z* parameter for the O1 atom is calculated using equation (8) and the calculated value is again consistent with those of the other phases
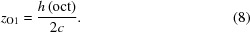
Various crystal structure data and calculated *d* spacings for the proposed model structure are given in Table 1 ▶. Comparison with Baneyeva and Popova’s structure show that it gives an improved match with the X-ray diffraction data and that the Zn—O distance is the same as that calculated using the effective ionic radii instead of being much longer, so the proposed structure is crystal-chemically more sensible. Baneyeva and Popova did not locate the H1 atom but a value for *z*_H1_ is given here for completeness. It is calculated using equation (9), which requires knowledge of the oxygen–hydrogen distance, *d*(O—H), which is set here at 1 Å – a value similar to those determined by neutron diffraction for Mg(OH)_2_ and Ca(OH)_2_ – but it of course needs to be determined by experiment.
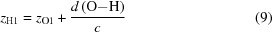
A schematic polyhedral representation of the new model structure for β-Zn(OH)_2_ is shown in Fig. 3 ▶(*a*).

## 5. Layered double hydroxides   

As noted in §1, LDH phases are derived from layered divalent metal hydroxides by the substitution of a fraction (*x*) of the divalent cations (*M* ^2+^) by trivalent cations (*M* ^3+^). This creates a +1 charge on the main layer and this positive charge is balanced by anions (*A* ^*n*−^) in the interlayer region, which also contains water molecules. *A* ^*n*−^ can be single anions (such as Cl^−^ or OH^−^), trigonal planar anions (CO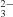, NO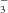), tetrahedral anions (SO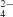, CrO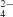), octahedral anions ([Fe(CN)_6_]^4−^) or alumino-silicate sheets (Drits & Bookin, 2001 ▶). Regarding octahedral anions, interesting work has been reported recently on phases with Sb(OH)_6_ octahedra in the interlayer (Bonaccorsi *et al.*, 2007 ▶; Mills, Christy, Kampf *et al.*, 2012 ▶; Mills, Kampf *et al.*, 2012 ▶). Whilst the interlayers of most LDH phases contain a single layer of anions and water molecules, more complex interlayer structures do occur (Evans & Slade, 2006 ▶; Mills, Christy, Genin *et al.*, 2012 ▶); for example, double layers of water molecules and anions, such as in Mg–Al sulfate LDH phases (Brindley & Kikkawa, 1980 ▶), and double layers of cations and anions, such as sodium and sulfate ions (Drits *et al.*, 1987 ▶), as in motukoreaite (Rius & Plana, 1986 ▶), magnesium and sulfate ions, as in mountkeithite (Hudson & Bussell, 1981 ▶), or calcium and sulfate ions, as in wermlandite (Rius & Allmann, 1984 ▶).

The general formula for LDH phases is
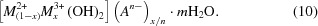
This formula is based on one main-layer cation; the contents of the main layer are within the square brackets. The number of water molecules per cation, *m*, is variable. The maximum value of *m* is given in equation (11) for monatomic anions or planar oxyanions that lie perpendicular to the **c** axis, where *N* is the number of interlayer sites that are occupied by the anion, *n* is the charge on the anion and the square symbol represents the fraction of interlayer sites that are not occupied; as an example, Taylor (1973 ▶) suggested a maximum value of 0.125 for the fraction of vacant interlayer sites in the pyroaurite polytypes (*i.e.* Mg—Fe—CO_3_ LDH phases).
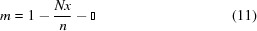



The mean effective ionic radius for the cation (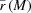) in LDH phases is reduced because of the replacement of a fraction (*x*) of the main-layer divalent cations by smaller trivalent cations. 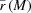 is calculated using equation (12)

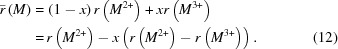
Equation (12) appears in many discussions in the literature concerning LDH phases – and is usually referenced to Brindley & Kikkawa (1979 ▶) – but unfortunately the right-hand side is often incorrectly equated with *d*(*M*—O) (*e.g.* in Leroux *et al.*, 2001 ▶; Intissar *et al.*, 2003 ▶; Forano *et al.*, 2006 ▶), including in two highly cited reviews (Cavani *et al.*, 1991 ▶; Evans & Slade, 2006 ▶); the error is the omission of *r*(OH^−^) from the equation [*cf.* equation (3)].

Substitution of 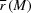 for 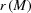 in equation (4) gives
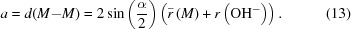
So
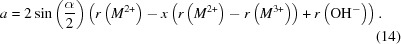
Expanding and rearranging equation (14) gives
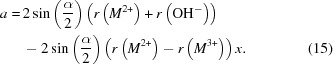
The first part of the right-hand side of equation (15) is equal to the *a* parameter of an *M*(OH)_2_-type phase, 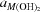 [*cf.* equation (4)], and so the *a* parameter for the layered double hydroxide, 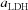, can be expressed as
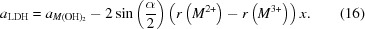
Equation (16) is essentially a statement of Vegard’s Law, *i.e.* that in a solid solution series there is a linear relation between the lattice constant and composition (Vegard, 1921 ▶; West, 1984 ▶; Denton & Ashcroft, 1991 ▶); deviations from Vegard’s Law are discussed by West (1984 ▶). Equation (16) has a negative slope
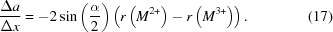
For regular octahedra α = 90° and substituting this into equation (14) gives
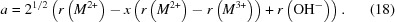
Therefore
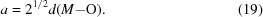
Substitution of α = 90° into equation (17) gives
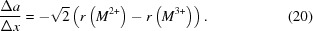
It is these two equations [(18)/(19) and (20)] that are widely reported in the literature when discussing the relationship between the composition of LDH phases, *x*, and the *a* parameter of the unit cell; for example, in papers concerned with Mg–Al LDH phases (Brindley & Kikkawa, 1979 ▶), Ni–Al (Brindley & Kikkawa, 1979 ▶), Zn–Al (Thevenot *et al.*, 1989 ▶), Mg–Ga (López-Salinas *et al.*, 1997 ▶), Co–Al (Leroux *et al.*, 2001 ▶), Mg–Al,Fe (Rozov *et al.*, 2010 ▶), and in the review articles by Cavani *et al.* (1991 ▶), Drits & Bookin (2001 ▶), and Evans & Slade (2006 ▶); there is a typographical error in Evans and Slade’s version of equation (19). Whilst the metal–oxygen octahedra in LDH phases are widely understood to be squashed – as in the layered single hydroxides – the discussions in the literature regarding the relationship between *x* and *a* have nevertheless not progressed since Brindley & Kikkawa’s (1979 ▶) seminal paper, *i.e.* the equations that are reported are always for regular octahedra [equations (18)/(19) and (20)]. It is not a surprise therefore that there have been few practical attempts to link *x* and *a*. Kooli *et al.* (1996 ▶) did calculate *x* from the *a* parameter for a series of Mg–Al LDH phases but they used an empirical relationship [the values of *x* and *a* in their Table 2 ▶ are not consistent with the use of equation (19) and the same group published a figure in a later paper that includes a linear regression fit; Kaneyoshi & Jones, 1999 ▶]. The calculation of *x* from *a* and *vice versa* using the geometrical approach requires the use of equation (16), and so accurate values are needed for α and 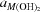 together with a satisfactory explanation for the value of the latter. As established earlier for the single hydroxides, the calculation of 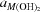 also requires an accurate value for the effective radius of the O atom of the hydroxyl ion, *r*(OH^−^(IV)) [*i.e.* for use in equation (15)], which is not necessarily the same in LDH phases as for the single hydroxides; for example, it will be affected by the strength of hydrogen bonding, which is different in single and double hydroxides.

## 6. Determination of *a*_*M*(OH)_2__ and α for layered double hydroxides   

**Figure 4:**
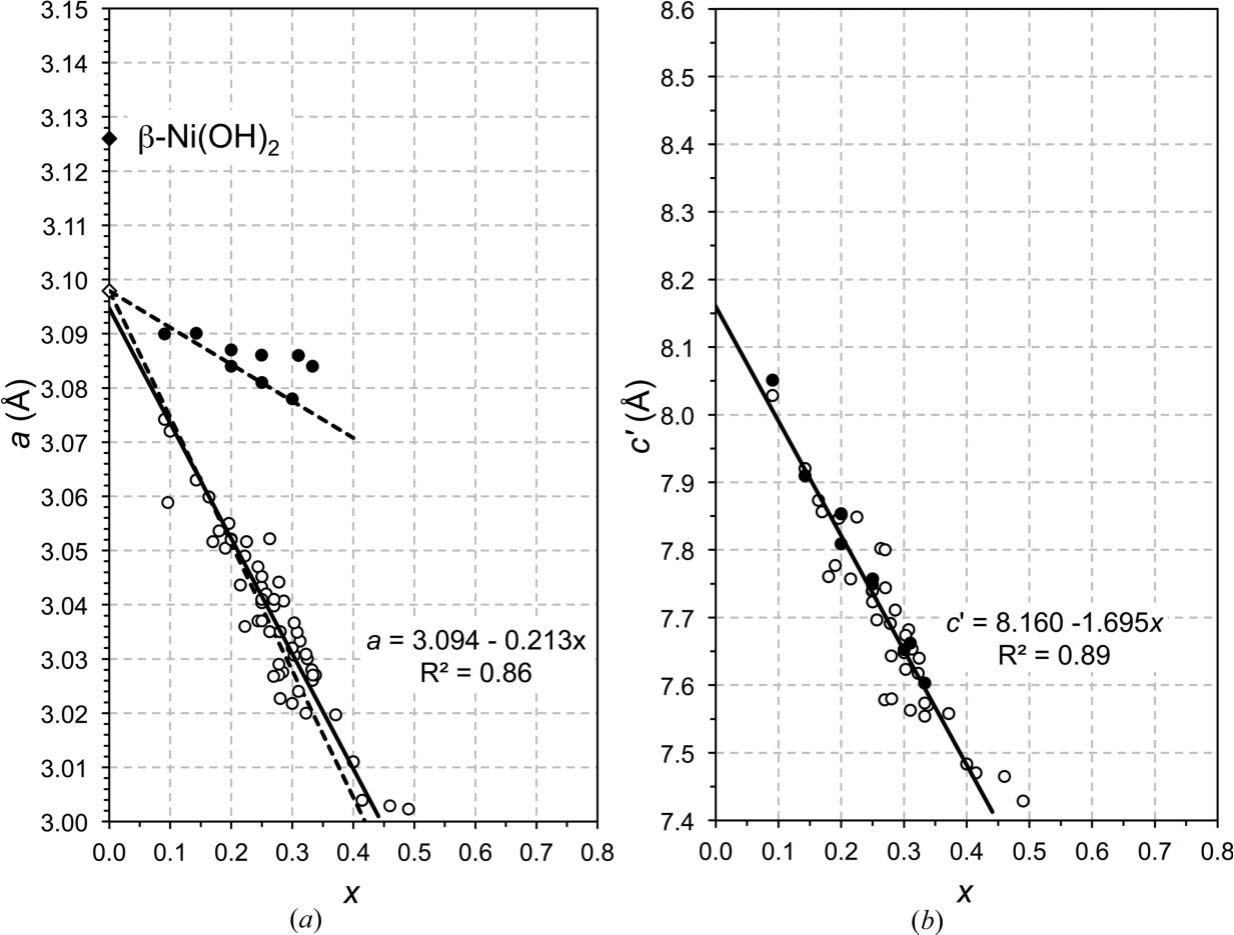
The lattice parameters of takovite-like phases. (*a*) The *a* parameter plotted against *x* for a range of Ni–Al (open circles) and Ni–Fe (filled circles) LDH preparations reported in the literature. The data for Ni–Al LDH phases are from the following references: Brindley & Kikkawa (1979 ▶); Carteret *et al.* (2011 ▶); d’Espinose de la Caillerie *et al.* (1995 ▶); Hu & Noréus (2003 ▶); Indira *et al.* (1994 ▶); Jinesh *et al.* (2010 ▶); Johnson & Glasser (2003 ▶); Kovanda *et al.* (2009 ▶); Pérez-Ramírez *et al.* (2001 ▶); Prevot *et al.* (2009 ▶); Rives *et al.* (2003 ▶); Sato *et al.* (1988 ▶); Tsuji *et al.* (1993 ▶); Vieira *et al.* (2009 ▶); del Arco *et al.* (1999 ▶); Han *et al.* (2009 ▶). (*b*) The *c*′ parameter plotted against *x* for Ni—Al—CO_3_ (open circles) and Ni—Fe—CO_3_ (filled circles) LDH phases reported in the literature. The data are from: Brindley & Kikkawa (1979 ▶); Carteret *et al.* (2011 ▶); d’Espinose de la Caillerie *et al.* (1995 ▶); Jinesh *et al.* (2010 ▶); Johnson & Glasser (2003 ▶); Kovanda *et al.* (2009 ▶); Prevot *et al.* (2009 ▶); Rives *et al.* (2003 ▶); Sato *et al.* (1988 ▶); del Arco *et al.* (1999 ▶); Han *et al.* (2009 ▶).

LDH phases with variable composition (*i.e.* variable *x*) but fixed types of di- and trivalent cations have been studied very widely. The first workers to relate the value of the *a* parameter extrapolated to *x* = 0 to the values for the corresponding β-*M*(OH)_2_ phases seem to have been Brindley & Kikkawa (1979 ▶) for Mg–Al and Ni–Al LDH systems and Miyata (1980 ▶) for Mg–Al. Brindley and Kikkawa considered that agreement was good for β-Mg(OH)_2_ (which occurs naturally as brucite) – as did Miyata – but not particularly good for β-Ni(OH)_2_ (theophrastite). They noted that only a small change in the slope of the plot of *a* against *x* would have a large effect on the extrapolated value for the lattice parameter of the *M*(OH)_2_ phase and thus considered that any lack of agreement between the extrapolated value for the *a* parameter and that measured directly for β-*M*(OH)_2_ phases is due to experimental errors. This explanation has been widely accepted (*e.g.* Drits & Bookin, 2001 ▶). Carteret *et al.* (2011 ▶) did state that ‘*the x = 0 compound is not the β-Ni(OH)_2_ phase*’ in a recent study of Ni–Al and Ni–Fe LDH preparations but they did not provide an alternative explanation. Their data are included with those of other workers in Fig. 4 ▶(*a*), which is a plot of *a* against *x* for a range of Ni–Al (open circle) and Ni–Fe (filled circles) LDH preparations. The full line is the result of the linear regression analysis of the Ni–Al LDH data and the filled diamond represents β-Ni(OH)_2_. It is apparent on inspection of Fig. 4 ▶(*a*) that the trends for both the Ni–Al and Ni–Fe series lead to a similar value of *a* at *x* = 0 and that the value established using those trends is not close to the *a* parameter of the β-Ni(OH)_2_ phase. Fig. 4 ▶(*b*) shows a plot of the layer spacing, *c*′, against *x* for Ni–Al (open circle) and Ni–Fe LDH (filled circle) preparations that have carbonate ions as the charge-balancing interlayer anions. The data all follow the same linear trend, regardless of whether the trivalent ion is Al or Fe. This shows that the reduction in layer spacing that occurs as *x* increases is due entirely to the increase in the electrostatic attraction between the negative interlayer and the positive hydroxide main layer. The full line on Fig. 4 ▶(*b*) is the result of linear regression analysis; the value of *c*′ at *x* = 0 is 8.16 Å. Since there are no trivalent cations when *x* = 0, this value of *c*′ corresponds to the situation where the main hydroxide layer is electroneutral and so there would be no anions in the interlayer. This interlayer spacing is very much greater than the value for β-Ni(OH)_2_ – which is 4.606 Å (Kazimirov *et al.*, 2010 ▶) – and so there must be something in the interlayer region that keeps the hydroxide layers so far apart. It seems reasonable to suggest that the expanded interlayer is due to the presence of water molecules, *i.e.* that the phase at *x* = 0 has essentially the same general structure as the LDH phase but with no trivalent cations or interlayer anions. From formula (10) and equation (11) the formula would be Ni(OH)_2_·H_2_O if all of the interlayer sites that are available for the O atoms of water molecules are occupied [*x* = 0 and □ = 0 in equation (11)]. It seems probable therefore that the phase at *x* = 0 is one of the so-called α forms of divalent metal hydroxides (Feitknecht, 1938 ▶), which are disordered and contain a variable amount of interlayer water. α-Ni(OH)_2_-type phases have been studied widely because of their relevance to batteries (*e.g.* Oliva *et al.*, 1982 ▶; Kamath *et al.*, 1994 ▶; Li *et al.*, 2006 ▶).

**Figure 5:**
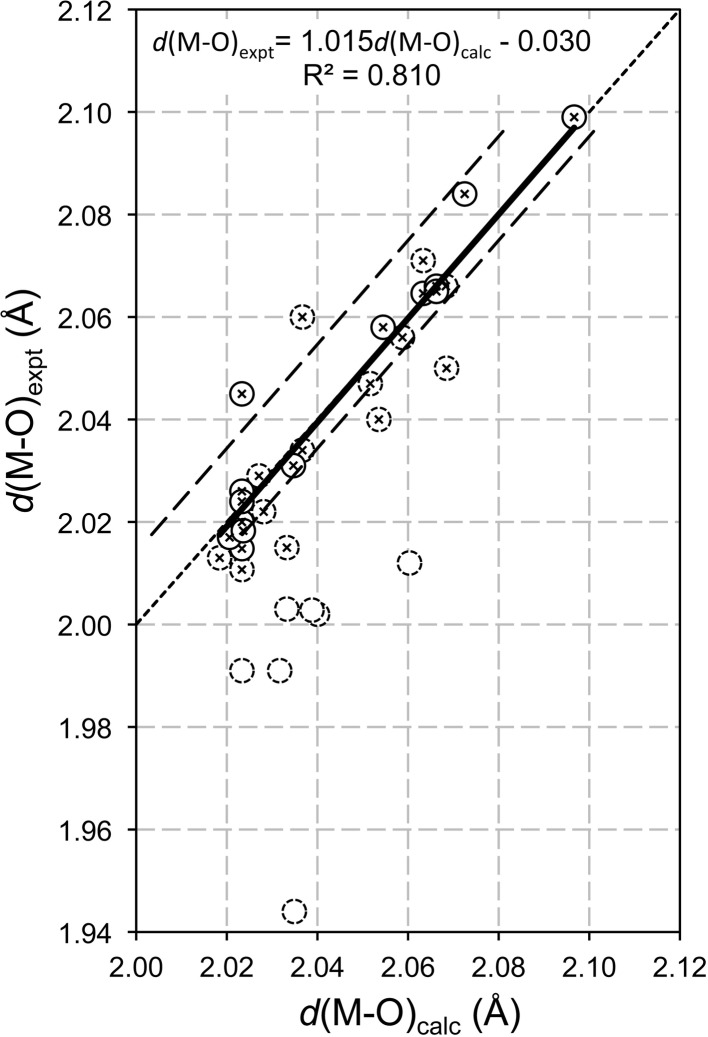
*d*(*M*—OH) determined by experiment for a variety of LDH phases plotted against the value calculated using ionic radii. The circles with full-line circumferences represent the data from structures that were determined using single crystals and the dotted-line circles are for structures determined using powder data. The lines are explained in the text. The data are from the following references: (1) For single crystals: Allmann (1968 ▶, 1969 ▶) (pyroaurite and ‘sjögrenite’); Arakcheeva *et al.* (1996 ▶) (quintinite-2*H*-3*c*); Braithwaite *et al.* (1994 ▶) (iowaite); Cooper & Hawthorne (1996 ▶) (shigaite); Huminicki & Hawthorne (2003 ▶) (nikischerite); Krivovichev *et al.* (2010*a* ▶) (quintinite-2*H*-3*c*); Krivovichev *et al.* (2010*b* ▶) (quintinite-1*M*); Merlino & Orlandi (2001 ▶) (zaccagnaite); Pastor-Rodriguez & Taylor (1971 ▶) (coalingite); Rius & Allmann (1984 ▶) (wermlandite); Rius & Plana (1986 ▶) (motukoreaite); Zhitova *et al.* (2010 ▶) (quintinite-2*H*); (2) for powder where the data are used in the fit: Bellotto *et al.* (1996 ▶) (Mg–Al); Constantino *et al.* (1998 ▶) (Mg–Al); Ennadi *et al.* (2000 ▶) (Zn–Al); Lombardo *et al.* (2005 ▶) (Zn–Al–Cl); Lozano *et al.* (2012 ▶) (Zn–Al, zaccagnaite-3*R*); Manohara & Vishnu Kamath (2010 ▶) (Co–Ga, Mg–Ga); Manohara *et al.* (2011 ▶) (Mg–Fe); Mills *et al.* (2011 ▶) (Mg–Cr, barbertonite); Radha *et al.* (2007*a* ▶) (Zn–Al); Roussel *et al.* (2000 ▶) (Zn–Cr); Witzke & Raade (2000 ▶) (Zn–Al, zincwoodwardite); (3) powder data outliers: Lombardo *et al.* (2005 ▶) (Zn–Al–CO_3_); Manohara & Vishnu Kamath (2010 ▶) (Ni–Ga); Mills *et al.* (2011 ▶) (Mg–Cr, stichtite); Radha *et al.* (2011 ▶) (Zn–Al–SO_3_, Zn–Al–IO_3_); Radha *et al.* (2007*b* ▶) (Co–Al, Mg–Al).

If the phase at *x* = 0 is an α nickel hydroxide, then its *a* parameter determined by experiment is given by the regression analysis equation in Fig. 4 ▶(*a*), *i.e.* 3.094 Å. As noted earlier, 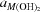 can be calculated using equation (15), which requires accurate values for α and the effective radius of the O atom of the hydroxyl ion, *r*(OH^−^(IV)). A value for *r*(OH^−^(IV)) for LDH phases can be established in the same way as for *r*(OH^−^(IV)) for the single hydroxides (Fig. 2 ▶ *b*), *i.e.* by plotting values of *d*(*M*—OH) determined by experiment for a variety of LDH phases against the values calculated from ionic radii using equation (3), but with 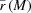 [calculated using equation (12)] substituted for *r*(*M* ^2+^). Such a plot is shown in Fig. 5 ▶. The dotted line in this figure represents perfect agreement between the calculated values and those determined by experiment. The full line shows that there is excellent agreement between the calculated and measured data when a value of *r*(OH^−^(IV)) = 1.365 Å is used in the calculation. The dashed line that is to the left of the full line shows the position of the fitted line when Shannon’s value for *r*(OH^−^(IV)) is used instead in the calculation (*i.e.* 1.35 Å) and the dashed line to the right is the position when the value established earlier for single hydroxides is used (*i.e.* 1.37 Å); both alternative values can be seen to be wrong. Only the data presented as circles with crossed centres were used in the fit, which includes all of the structures that were determined using single crystals because they might be expected to be more reliable than those determined using powder. Comparison of Fig. 5 ▶ with Fig. 2 ▶(*b*) shows that the data for LDH phases do not produce a linear correlation that is as strong as for the single hydroxides and this would seem to suggest that some of the structure determinations for LDH phases ought to be revisited. The values of *d*(*M*—OH)_expt_ for the structures of the outlying data points should be considered unreliable, or alternatively the reported compositions of the samples are wrong, which would result in mistakes in the calculation of *d*(*M*—OH)_calc_. It is evident that the calculation used here can be used as a sanity test for structure determinations of LDH phases.

Substitution of the value established here for *r*(OH^−^(IV)) (*i.e.* 1.365 Å) together with the ionic radius for Ni^2+^(VI) (from Table 1 of Shannon, 1976 ▶) into equation (15) allows a value for α to be derived by adjusting it to give the best match with the linear regression line for Ni—Al—CO_3_ LDH data in Fig. 4 ▶(*a*). The result is shown by the dashed line on Fig. 4 ▶(*a*) that falls close to the regression line (which is the full line); the value of α obtained in this way is 97.83°. The *a* parameter for this α-Ni(OH)_2_ phase is obtained by substituting *r*(OH^−^(IV)) = 1.365 Å and α = 97.83° into equation (4). This gives 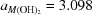
 Å (represented by an open diamond in Fig. 4 ▶ *a*), which is very close to the value from the regression analysis of the Ni–Al data (*i.e.* 3.094 Å). The upper dashed line in Fig. 4 ▶(*a*) is the line calculated for Ni—Fe—CO_3_ LDH phases using the same values for *r*(OH^−^(IV)) and α, *i.e.* repeating the calculation but exchanging *r*(Fe^3+^(HS)(VI)) for *r*(Al^3+^(VI)) (values from Shannon, 1976 ▶). It is evident that this simple calculation has resulted in excellent agreement with experimental data for both Ni—Al—CO_3_ and Ni—Fe—CO_3_ LDH preparations. The value of α appears to be independent of the type of trivalent cation.

Some of the data points for the Ni—Fe—CO_3_ LDH preparations are above the upper dashed line in Fig. 4 ▶(*a*), which suggests that there is a problem with those data: perhaps an error in the composition, the *a* parameter or both. The value of *x* could be wrong either because the composition of the initial mix used in the synthesis procedure was simply assumed to apply to the solid and was not checked, or because of incorrect interpretation of the bulk analysis if the preparation contained more than one phase. Since second phases in LDH preparations can be amorphous or crystalline it may be necessary to measure the actual composition of the LDH crystals, for example by microanalysis in a transmission electron microscope. Errors can occur in the measurement of the *a* parameter, particularly where the (110) peak on the X-ray diffraction pattern is broadened due to a small average crystal size [*a* is calculated from the *d*-spacing of the (110) peak, *a* = 2 × *d*_110_], which is of course why the proven link between *a* and *x* given in this paper provides an important check.

**Figure 6:**
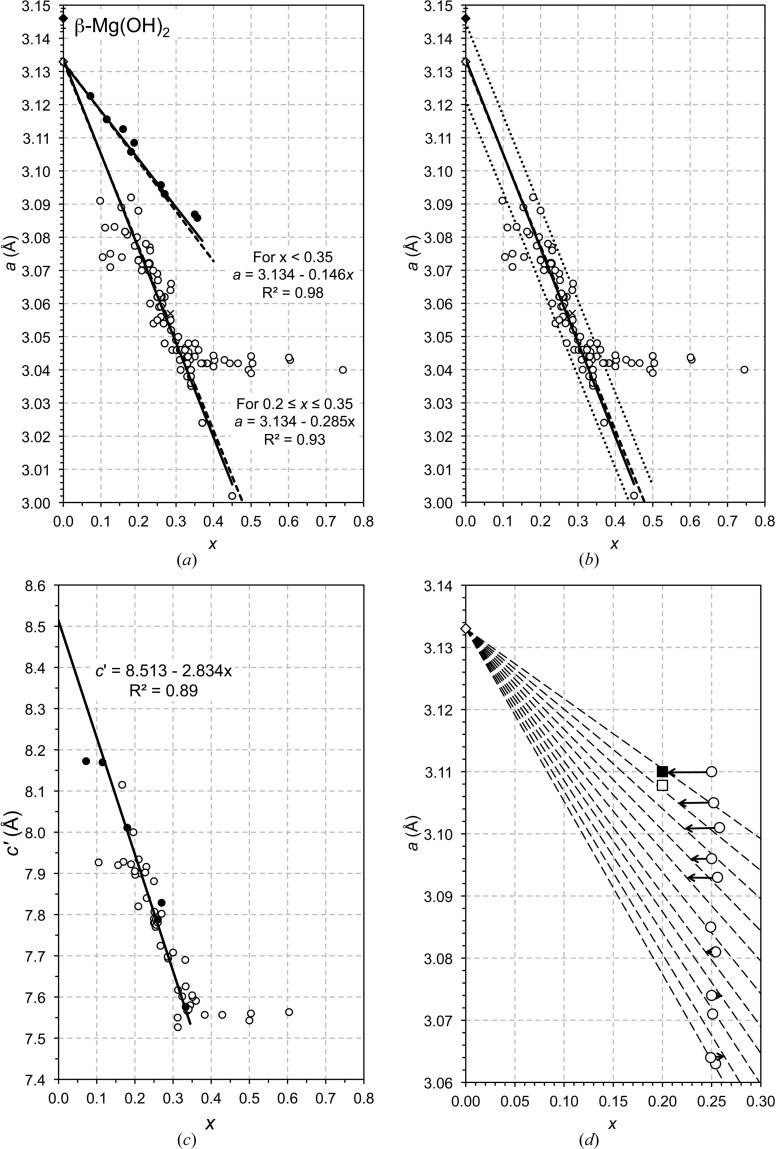
The lattice parameters of hydrotalcite-like phases. (*a*) The *a* parameter plotted against *x* for a range of Mg–Al (open circles) and Mg–Ga (filled circles) LDH preparations reported in the literature; the data for the Mg–Al LDH involve a variety of interlayer anions (*i.e.* OH^−^, CO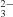, NO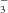, Cl^−^) and are from: Mg–Al: Bellotto *et al.* (1996 ▶); Bîrjega *et al.* (2005 ▶); Brindley & Kikkawa (1979 ▶); Budhysutanto *et al.* (2011 ▶); Gastuche *et al.* (1967 ▶); Han *et al.* (1998 ▶); Jinesh *et al.* (2010 ▶); Kaneyoshi & Jones (1999 ▶); Kukkadapu *et al.* (1997 ▶); Mascolo & Marino (1980 ▶); Miyata (1980 ▶); Pausch *et al.* (1986 ▶); Rao *et al.* (1998 ▶); Sato *et al.* (1988 ▶); Shen *et al.* (1994 ▶); Valente *et al.* (2011 ▶); Xu & Zeng (2001 ▶); Yun & Pinnavaia (1995 ▶); Mg–Ga: Bellotto *et al.* (1996 ▶); López-Salinas *et al.* (1997 ▶). (*b*) The Mg–Al data from Fig. 6 ▶(*a*) together with two dotted lines that represent the best value of α ± 0.5%. This corresponds to a difference in α of only 1°, *i.e.* the dotted lines are for α = 96.92 and 97.89°. (*c*) The *c*′ parameter plotted against *x* for a range of Mg–Al–CO_3_ (open circles) and Mg–Ga–CO_3_ (filled circles) LDH preparations reported in the literature. Data are from: Mg–Al: Bellotto *et al.* (1996 ▶); Bîrjega *et al.* (2005 ▶); Brindley & Kikkawa (1979 ▶); Budhysutanto *et al.* (2011 ▶); Gastuche *et al.* (1967 ▶); Iyi *et al.* (2007 ▶); Iyi & Sasaki (2008 ▶); Jinesh *et al.* (2010 ▶); Miyata (1980 ▶); Pausch *et al.* (1986 ▶); Rao *et al.* (1998 ▶); Sato *et al.* (1988 ▶); Shen *et al.* (1994 ▶); Valente *et al.* (2011 ▶); Yun & Pinnavaia (1995 ▶); Mg–Ga: Bellotto *et al.* (1996 ▶); López-Salinas *et al.* (1997 ▶). (*d*) The *a* parameter plotted against *x* for Mg–Al,Fe LDH preparations. The lines are explained in the text. The data are from Rozov *et al.* (2010 ▶) (open circle), Manohara *et al.* (2011 ▶) (open square) and Meng *et al.* (2004 ▶) (filled square).

Fig. 6 ▶(*a*) is a plot of the *a* parameter against *x* for a range of Mg–Al (open circle) and Mg–Ga (filled circle) LDH preparations reported in the literature; the data for the Mg–Al LDH involve a variety of interlayer anions (*i.e.* OH^−^, CO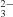, NO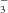, Cl^−^). The full lines are the result of the linear regression analyses of both sets of data; since the solid solution occurs over a limited range of *x* (the reason for this limited range is discussed by Brindley & Kikkawa, 1979 ▶) the fit for the Mg–Al data is for 0.2 ≤ *x* ≤ 0.35. The figure includes points for the β polymorph of magnesium hydroxide (*i.e.* brucite, filled diamond) and a theoretical α form (open diamond), in this case a proposed hydrated form, Mg(OH)_2_·H_2_O. A solid solution end-member with this composition was actually suggested over 40 years ago by Taylor (1969 ▶) in a study of the Mg–Fe LDH phases pyroaurite and ‘sjögrenite’ (sjögrenite has been discredited recently as a separate mineral species: it is the 2*H* polytype of pyroaurite (Mills, Christy, Genin *et al.*, 2012 ▶). The *a* parameter for the α-Mg(OH)_2_ phase, which is calculated as above for the Ni-based system, is the same as the value from the regression analysis of both sets of data. The dashed lines represent the values of *a* calculated using equation (15); the value of α is in this case 97.41°. It is clear that the calculated lines are strikingly similar to those from the regression analysis of the experimental data, which supports strongly the geometrical approach developed in this paper, together with the values determined for *r*(OH^−^(IV)) and the bond angle α. The same value of α is again appropriate with either trivalent ion, in agreement with the observation for the Ni-based systems; *i.e.* the extent to which the metal–oxygen octahedra are squashed is independent of the type of trivalent ion in both Ni- and Mg-based systems. The exact positions of the calculated (*i.e.* dashed) lines on Figs. 4 ▶(*a*) and 6 ▶(*a*) are affected by the values of both *r*(OH^−^(IV)) and the bond angle α, which is the reason why a value for *r*(OH^−^(IV)) was determined using Fig. 5 ▶. The positions of the lines as shown were as a consequence established simply by adjusting the value of α, and they are in fact rather sensitive to it. This is illustrated in Fig. 6 ▶(*b*), which shows the Mg–Al data from Fig. 6 ▶(*a*) together with two dotted lines that represent the best value of α ± 0.5%, which corresponds to a difference in α of only 1° (*i.e.* the dotted lines are for α = 96.92 and 97.89°).

Fig. 6 ▶(*c*) is a plot of the layer spacing, *c*′, against *x* for the Mg–Al (open circle) and Mg–Ga (filled circle) LDH preparations where the charge-balancing interlayer anions are carbonate ions. As for the Ni-based preparations, the data all follow the same linear trend, regardless of whether the trivalent ion is Al or Ga. This again shows that the reduction in layer spacing that occurs as *x* increases is due entirely to the increase in the electrostatic attraction between the negative interlayer and the positive hydroxide main layer. The linear regression analysis – which is shown by the full line – indicates a value of *c*′ = 8.51 Å at *x* = 0. Again, as with the Ni-based system, it is proposed here that this value corresponds to the layer spacing of an α magnesium hydroxide that will have the formula Mg(OH)_2_·H_2_O if all of the interlayer sites that are available for the O atoms of water molecules are occupied.

## 7. Substitution by two different types of trivalent cations   

It is evident from the foregoing discussion that for a given value of *x* the replacement of aluminium by another trivalent ion would result in a different *a* parameter but the same value of *c*. The treatment is easily modified to allow for the incorporation of more than one type of trivalent ion, for example a mixture of Al^3+^ and Fe^3+^. 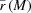 is simply calculated using equation (21) instead of equation (12)

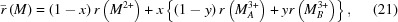
where *x* is still the fraction of cations that are trivalent, and *y* is the fraction of those trivalent ions that are of a second type; so 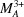 would be one type, say Al^3+^, and 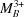 the other, say Fe^3+^. Equation (15) is thus modified to equation (22)

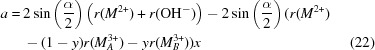
and so
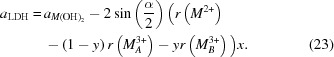
It was established earlier that the trivalent cation has no effect on the value of α, which is determined entirely by the type of divalent cation and there is no reason to suppose that the situation is any different in systems that have two types of trivalent ion. Rozov *et al.* (2010 ▶) report data for Mg-based LDH preparations that have bulk *x* ≃ 0.25 and varying amounts of Al^3+^ and Fe^3+^; their data are presented in Fig. 6 ▶(*d*) (open circle). The dashed lines on the figure represent equation (23) using Rozov *et al.*’s values of *y* (with *y* set as the fraction of trivalent ions that are Fe^3+^) and the value for α that was determined earlier for Mg-based preparations (*i.e.* 97.41°). The top dashed line corresponds to *y* = 1 in equations (22) or (23) (*i.e.* no Al) and the lines below it are at *y* intervals of approximately 0.1. The bottom dashed line is the same as the dashed line in Fig. 6 ▶(*a*) (*i.e.* no Fe, *y* = 0). Inspection of Fig. 6 ▶(*d*) shows that Rozov *et al.*’s data points for 0 ≤ *y* ≤ 0.5 are very close to the values calculated using equation (23), but the points for 0.6 ≤ *y* ≤ 1 are not close (the difference between the experimental and theoretical positions are indicated by arrows). For comparison, there are two other data points plotted for Al-free preparations, which – in contrast to Rozov *et al.*’s data – are close to the theoretical position. Those data are from Meng *et al.* (2004 ▶) (filled square) and Manohara *et al.* (2011 ▶) (open square). The fact that Rozov *et al.*’s data for 0 ≤ *y* ≤ 0.5 and Meng *et al.*’s and Manohara *et al.*’s for *y* = 1 are consistent with the calculated values is further support for the utility of the treatment described in this paper, and suggests that the compositions for Rozov *et al.*’s samples with 0.6 ≤ *y* ≤ 1 are possibly in error. It is possible that this could be due to the presence of an undetected second phase since Rozov *et al.* determined the composition of their solids using a bulk technique. As noted earlier, since second phases present in LDH preparations can be amorphous as well as crystalline it would be useful to check the composition of the LDH crystals by microanalysis in a transmission electron microscope.

The evidence presented in Fig. 6 ▶(*d*) suggests that there is a problem with the chemical composition assumed by Allmann (1968 ▶) for pyroaurite, *i.e. x* = 0.25. In that study the *a* parameter is reported to be 3.113 Å, which thus corresponds to a position on the figure above the highest point of Rozov *et al.*’s data. The composition assumed by Allmann would therefore appear to be in error with a value for *x* of 0.18 more likely. The evidence also indicates that the *a* parameter reported by Ingram & Taylor (1967 ▶) for pyroaurite is also likely to be incorrect (3.13 Å, which is much too high).

## 8. Model crystal structures for layered double hydroxides   

The crystal-chemical treatment described in this paper and the extent of the data collated in Figs. 4 ▶ and 6 ▶ mean that model structures that are crystal-chemically sensible can be provided for Ni- and Mg-based LDH phases that have any value of *x* (that is consistent with experiment), seemingly any trivalent cation, and that have carbonate as the charge-balancing interlayer ion; *i.e.* phases that have the general formula (24), where *M* ^2+^ is Ni^2+^ or Mg^2+^. In principle, the same approach is possible for other divalent metals, but there are currently insufficient data available in the literature to allow prediction of the *c* parameter
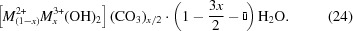
Natural and synthetic LDH phases usually occur in structures that involve either a two- or three-layer stacking sequence of the layers. Bookin and Drits showed that there are three possible two-layer polytypes, each of which has hexagonal stacking of the layers (denoted 2*H*_1_, 2*H*_2_ and 2*H*_3_), and a total of nine three-layer polytypes, of which two have rhombohedral symmetry (3*R*_1_ and 3*R*_2_); the other seven three-layer polytypes have hexagonal symmetry (denoted 3*H*_1_ to 3*H*_7_; Bookin & Drits, 1993 ▶; Bookin *et al.*, 1993 ▶; Drits & Bookin, 2001 ▶). Detailed discussion of the different polytypes and notation can be found in Bookin & Drits (1993 ▶), Drits & Bookin (2001 ▶), Evans & Slade (2006 ▶) and Mills, Christy, Genin *et al.* (2012 ▶). LDH minerals are known in both 2*H* and 3*R* forms; for example ‘manasseite’ and ‘sjögrenite’ are the 2*H*_1_ polytypes of hydrotalcite and pyroaurite respectively, which are 3*R*_1_ polytypes (Mills, Christy, Genin *et al.*, 2012 ▶). Synthetic LDH phases are usually 3*R*_1_, which is the polytype structure that is used here.

The positions of the atoms in the asymmetric unit of such a model structure are given in Table 2 ▶. The structure is based on Allmann & Jepsen’s (1969 ▶) structure for hydrotalcite (space group 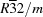, No. 166, hexagonal axes), although many other derivative structures have been published that involve various cations and interlayer anions (*e.g.* Bellotto *et al.*, 1996 ▶; Constantino *et al.*, 1998 ▶; de Roy *et al.*, 2001 ▶; Radha *et al.*, 2007*a* ▶,*b* ▶; Johnsen *et al.*, 2010 ▶; Manohara *et al.*, 2011 ▶; Mills *et al.*, 2011 ▶). O1 is the O atom of the octahedral layer and O2 belongs to the interlayer anion or water molecule. The H atoms of interlayer water are not included. The values of *z*/*c* for the O1 and H1 atoms (*i.e. z*_O1_ and *z*_H1_) depend on the fraction of *M* ^3+^ ions (*i.e.* on ‘*x*’) and are calculated using equations (25) and (9), respectively; equation (9) requires knowledge of the oxygen–hydrogen bond distance, *d*(O—H). For the purpose of these model structures *d*(O—H) is set at 1 Å and *x*_O2_ at 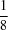. *z*/*c* is the distance from the metal ion to the planes of O atoms (*i.e.* the distance *M*—P on Fig. 1 ▶)
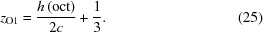

*h*(oct) is the thickness of the main layer and is calculated using equation (6), but with 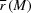 substituted for 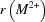; *h*(oct) is indicated in Fig. 3 ▶(*b*). 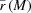 is calculated using equation (12) if there is one type of trivalent cation, or equation (21) if there are two types.

The *a* parameter for a model structure is calculated using equations (15) or (22) – depending on whether there is one type of trivalent cation or two – using Shannon’s values for *r*(*M* ^*n*+^(VI)) and those determined in this paper for *r*(OH^−^(IV)) and α; *i.e.* α is 97.83° for Ni-based phases and 97.41° for Mg-based, and *r*(OH^−^) is 1.365 Å for both. The *c* parameter is calculated using the relevant regression analysis equation that is given in Figs. 4 ▶(*b*) or 6 ▶(*c*) (*i.e.* for a three-layer polytype *c* = 3 × *c*′). As examples, Table 3 ▶ gives crystal-structure data for model structures that have *x* = 0 and 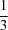, *i.e.* *M*(OH)_2_·H_2_O and [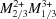
(OH)_2_](CO_3_)_1/6_·½H_2_O], where *M* ^2+^ is Ni^2+^ or Mg^2+^ and *M* ^3+^ is Al^3+^. *h*(inter) is the interlayer separation, *h*(inter) = *c*′ − *h*(oct). *V*_cell_ and *V*_oct_ are the volumes of the unit cell and the *M*—O octahedron respectively. *M*_r_ is the formula mass. *D*_c_ is the maximum calculated density (which can be calculated for any permitted composition); vacant interlayer sites would of course result in lower values. The structures are illustrated in Figs. 3 ▶(*b*) and (*c*), which are specifically for the Ni-based phases but the Mg-based look essentially the same at the scale used. For illustrative purposes the carbonate ions are shown ordered (associated with the Al ions).

Table 3 ▶ includes a model structure for an α form of Ni(OH)_2_. The interlayer has water molecules but no anions and all of the oxygen sites in the interlayer are occupied: the composition is Ni(OH)_2_·H_2_O and the calculated density is 2.71 g cm^−3^. If one third of the oxygen sites in the interlayer were vacant, the composition would obviously be [Ni(OH)_2_]·2/3H_2_O, and the density would be 2.56 g cm^−3^. This is essentially the same value that was reported by Bode *et al.* (1966 ▶) as the measured density of an α-nickel hydroxide that had the same composition. Bode *et al.* proposed a structure that gave a similar calculated density but inspection reveals that it is unrealistic because the Ni—O distance is much too long [2.23 Å, which implies a value of 1.54 Å for *r*(OH^−^), which is implausible] and the octahedra are not squashed (α is 87.31°).

## 9. Summary   

The importance and utility of proper crystal-chemical and geometrical reasoning in structural studies has been demonstrated through the consideration of layered single and double hydroxides. The approach has yielded new yet fundamental information on LDH phases and it is evident that their crystal chemistry is much more straightforward than is apparent from the literature. Equations have been derived that enable calculation of the composition (*x*) from the *a* parameter of the unit cell of LDH phases and *vice versa*, which can be used as a sanity test for extant and future structure determinations and computer simulation studies. Improved values have been determined for the effective radius of the O atom of the hydroxyl ion in both layered single and double hydroxides. The phase at *x* = 0 on *a*–*x* and *c*′–*x* plots for LDH phases is shown to be an α-*M*(OH)_2_ phase rather than the β polymorph. The extent to which the metal–oxygen octahedra in the main layer of LDH phases are squashed is shown to be independent of the type of trivalent ion, which also has no substantial effect on the reduction in the layer spacing that occurs as *x* increases. Model structures that are crystal-chemically sensible are given for Mg- and Ni-based LDH phases – including α-*M*(OH)_2_ phases – and also for β-Zn(OH)_2_.

*Note added in proof*: A single-crystal structure determination for takovite was published subsequent to the submission of the manuscript for this paper (Mills, Whitfield, Kampf *et al.*, 2012 ▶). The structure is very similar to the model structure that is given in Table 3 ▶ for the takovite-like phase with *x* = 0.333 and it has a value for the O—*M*—O angle α (97.86°) that is strikingly close to the value determined in Fig. 4 ▶(*a*) (97.83°), which is further support for the approach developed in this paper.

## Figures and Tables

**Table:** The experimental X-ray data, *d*_exp_, are from Baneyeva Popova (1969 ▶). The *d*-spacings calculated from Baneyeva and Popova’s structure and the structure derived in this work are given in the columns *d*_cBan_ and *d*_c1_, respectively. The space group is 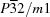 (No. 164) and the atom positions (*x*/*a*, *y*/*b*, *z*/*c*) in the asymmetric unit of both structures are Zn1: 0, 0, 0 and O1: 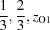. The position of the H1 atom is 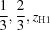. The value of *z*_O1_ which is calculated using equation (8) is given in the table (it is different for the two structures). *V*_cell_ and *V*_oct_ are the volumes of the unit cell and the ZnO octahedron, respectively.

No.	*hkl*	*d*_exp_ ()	*d*_cBan_ ()	*d*_c1_ ()
1	001	4.69	4.71	4.69
2	010	2.73	2.77	2.75
3	011	2.35	2.39	2.37
4	002	2.31	2.36	2.35
5	012	1.77	1.79	1.79
6	110	1.59	1.60	1.59
7	111	1.50	1.51	1.51
8	020	1.38	1.38	1.38
9	021	1.32	1.33	1.32
10	022	1.19	1.19	1.19
11	014	1.07	1.08	1.08
12	023	1.03	1.04	1.03
13	121	1.01	1.02	1.02

**Table:** 

	Baneyeva	This work
*a* ()	3.194	3.180
*c* ()	4.714	4.690
*V*_cell_ (^3^)	41.648	41.073
*z*_O1_	0.28	0.2217
*z*_H1_		0.4349
*V*_oct_ (^3^)	15.548	12.141
*d*(ZnO) ()	2.268	2.110
*h*(oct) ()	2.641	2.080
()	89.52	97.80

**Table 2:** The positions of the atoms in the asymmetric unit of a model structure for layered double hydroxide phases The space group is 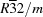 (No. 166, hexagonal axes). The ‘*x*’ in the occupancy (Occ.) column refers to the fraction of *M* ^3+^ ions in the formula rather than to the axial vector.

Label	Atom	Position	*x*/*a*	*y*/*b*	*z*/*c*	Occ.
*M*1	*M* ^2+^	3*a*	0	0	0	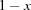
*M*2	*M* ^3+^	3*a*	0	0	0	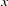
O1	O	6*c*	0	0	*z*_O1_	1
H1	H	6*c*	0	0	*z*_H1_	1
O2	O	18*h*	*x*_O2_	*x*_O2_	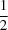	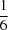
C1	C	6*c*	0	0	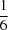	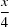

**Table 3:** Crystal-structure data for takovite- and hydrotalcite-like phases In all cases *x*_O2_ = 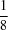 and *Z* = 3; other details are given in the text.

	Ni(OH)_2_H_2_O	[Ni_0.667_Al_0.333_(OH)_2_](CO_3_)_0.167_0.5H_2_O	Mg(OH)_2_H_2_O	[Mg_0.667_Al_0.333_(OH)_2_](CO_3_)_0.167_0.5H_2_O
	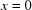	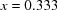	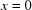	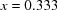
*a* ()	3.098	3.020	3.133	3.040
*c* ()	24.480	22.785	25.539	22.705
*V*_cell_ (^3^)	203.47	179.97	217.10	181.72
*z*_O1_	0.3747	0.3766	0.3739	0.3777
*z*_H1_	0.4155	0.4205	0.4131	0.4217
*V*_oct_ (^3^)	11.214	10.389	11.743	10.750
*d*(*M*O) ()	2.055	2.003	2.085	2.024
*h*(oct) ()	2.024	1.973	2.074	2.013
*h*(inter) ()	6.136	5.622	6.439	5.555
*M*_r_ (gmol^1^)	110.72	101.15	76.335	78.221
*D*_c_ (gcm^3^)	2.711	2.800	1.752	2.144
Wt% H_2_O	32.54	26.72	47.20	34.55
